# A general concept for consistent documentation of computational analyses

**DOI:** 10.1093/database/bav050

**Published:** 2015-06-08

**Authors:** Peter Ebert, Fabian Müller, Karl Nordström, Thomas Lengauer, Marcel H. Schulz

**Affiliations:** ^1^Computational Biology and Applied Algorithmics, Max Planck Institute for Informatics, Saarbrücken, Germany ^2^Graduate School of Computer Science, Saarland University, Saarbrücken, Germany ^3^Department of Genetics, Saarland University, Saarbrücken, Germany and ^4^Cluster of Excellence on Multimodal Computing and Interaction, Saarland University, Saarbrücken, Germany

## Abstract

The ever-growing amount of data in the field of life sciences demands standardized ways of high-throughput computational analysis. This standardization requires a thorough documentation of each step in the computational analysis to enable researchers to understand and reproduce the results. However, due to the heterogeneity in software setups and the high rate of change during tool development, reproducibility is hard to achieve. One reason is that there is no common agreement in the research community on how to document computational studies. In many cases, simple flat files or other unstructured text documents are provided by researchers as documentation, which are often missing software dependencies, versions and sufficient documentation to understand the workflow and parameter settings. As a solution we suggest a simple and modest approach for documenting and verifying computational analysis pipelines. We propose a two-part scheme that defines a computational analysis using a Process and an Analysis metadata document, which jointly describe all necessary details to reproduce the results. In this design we separate the metadata specifying the process from the metadata describing an actual analysis run, thereby reducing the effort of manual documentation to an absolute minimum. Our approach is independent of a specific software environment, results in human readable XML documents that can easily be shared with other researchers and allows an automated validation to ensure consistency of the metadata. Because our approach has been designed with little to no assumptions concerning the workflow of an analysis, we expect it to be applicable in a wide range of computational research fields.

**Database URL:** http://deep.mpi-inf.mpg.de/DAC/cmds/pub/pyvalid.zip

## Introduction

Large national and international research consortia like ICGC (https://icgc.org), DEEP (www.deutsches-epigenom-programm.de), Blueprint (www.blueprint-epigenome.eu) or ENCODE (1) generate and host vast amounts of genetic and epigenetic data. Thorough documentation and annotation is required on the side of the data provider in order to enable researchers from all over the world to access and process these datasets. The annotation metadata related to each datum ideally consist of concise descriptions of how individual files are generated. This description typically includes information on the procedures for sample acquisition, sample and donor characteristics such as health status, the type of assay and associated experimental protocols and details on the computer programs applied to analyse the resulting data. The latter item is usually limited to information on software name and version as well as basic parameter settings. The data avalanche that came with the rise of microarray and next-generation sequencing (NGS) technologies demanded the setup of high-throughput computational analysis tools and pipelines. Employing these pipelines typically results in a set of genome-scale measurements or annotations. The large number of results prohibits any manual evaluation and requires well-structured access to additional information to gather new biological insights. The scientific community has thus acknowledged the need for proper data curation and description. Coordinated efforts such as the ones undertaken by the International Society for Biocuration (2, 3) have been initiated to curate biological data and make them computationally available to research groups. Additionally, several format specifications have been developed to comprehensively capture the handling of biological samples in complex studies. These formats are either tailored to specific assays, such as MAGE-TAB (4) for microarrays, or are more generally applicable like the MAGE-TAB based BIR-TAB specification developed by the modENCODE consortium (5). The ISA-TAB (6) specification does not only link biological samples to protocols and derived data, it also allows to describe complex investigations encompassing several individual studies, each one in turn consisting of a number of assays. However, while these examples provide solutions to describe study setups in combination with experimental protocols, they have not been designed to document computational analyses consistently and in all detail, as they do not include templates to record the individual steps of an analysis.

Apart from curation efforts and consistent record keeping, due to continuously improved and updated annotations of biological entities such as reference genome assemblies and gene models [e.g. GENCODE (7)], proper versioning of data descriptions has become crucial. Ideally, i.e. when all data and metadata for a particular study are available in a versioned and standardized format, this would enable independent researchers to reproduce the results, provided the respective software environment. In software development, version control systems like Subversion (https://subversion.apache.org) or git (http://git-scm.com) have proven useful for keeping track of changes in program code. For biological data, the overall pace of change is slow compared with the rapid cycles in software development. The high rate of change in software development is due to the multitude of motivations for altering program code: fixing a bug, replacing an algorithm with a better one, changing the control flow in the program or using a more appropriate data structure, to name just a few. Despite all these reasons for changing software, good programmers aim for high stability and robustness of their software interface, e.g. the naming of command line parameters should not change with an incremental software update. This is a vital property of a software to be used in a production environment, yet it lures the user into believing that ‘nothing critical changed’ after installing an update.

As exemplified in the Motivation, these principles are not generally applied to documenting and describing the processing of publically available biological datasets, potentially due to much lower rates of change with such data.

In this work, we describe a concept for making metadata on computational analysis pipelines available that respects the characteristics outlined earlier.

## Motivation

To argue why a standardized approach to the description of computational analyses is beneficial to the scientific community, let us consider the example of an arbitrary ENCODE ChIP-seq experiment (for instance ENCODE accession ENCSR000AKA; GEO sample accession GSM733708) and focus on the histone peak file. The description page (http://genome.ucsc.edu/cgi-bin/hgFileUi?db=hg19&g=wgEncodeBroadHistone) for this track lists information concerning the short-read alignment and the subsequent peak calling in a free text paragraph. The alignment of the data against the hg19 reference genome (8) was performed using MAQ (http://maq.sourceforge.net) with two non-default parameters, yet no software version is given. The next step (filter 1), filtering out reads with more than ten best matches in the genome, is just described in plain text, presumably because no special software is necessary to perform the filtering. The subsequently applied peak calling tool is Scripture (9), again no information about the software version is included (we note that in the summary page for all tracks of that sample, the ‘Lab specific informatics’ field holds the information ‘ScriptureVPaperR3’, but we cannot connect that name to any version of Scripture). The Scripture command line is again described in plain text, stating parameters -task chip and -windows as well as informing that neither -trim nor a ‘mask file’ have been used. This form of describing a command line forces the user to refer to the tool documentation for more specific information; hence, it is laborious to assemble an actually executable command line for Scripture reproducing the results. The track description lists one more filtering step (filter 2), which has been implemented in MATLAB (MATLAB and Statistics Toolbox Release 2012b, The MathWorks, Inc., Natick, MA, USA), but gives no more specifics, in particular the MATLAB code is not available. All in all, the reproducibility of the results heavily relies (i) on the user’s experience in executing the described tools with correct parameter settings, (ii) on the identification and availability of a tool version that does not generate a different output despite identical parameter settings, and, in particular, (iii) on performing the two unspecified filtering steps (filter 1 and 2 in the above text) in exactly the same way. The only alternative is to contact the developer of the software or the corresponding researcher responsible for the analysis and ask for all computational sources to run the analysis—a procedure that might prove difficult if this contact person has left the institute.

This case example illustrates how, even in large, reputable consortia, the annotation of computational analyses is usually not on par with the level of detail one can find in metadata describing biological procedures or entities. This situation does not only impede the reproducibility of results, but also affects future studies that aim for comparability to existing data by implementing supposedly identical wet and dry lab protocols.

We propose an alternative form of computational metadata specification that allows for a complete record of all computational operations applied to a file with reasonable effort on the developer’s side. Our new approach is based on the following central requirements:

- results in human readable documents
- uses simple and established data formats
- is flexible enough to describe various types of computational analyses
- is a structured way of describing analyses
- includes version information
- allows for automatic generation of most metadata
- allows for automatic validation and consistency checks
- is lightweight enough to allow easy sharing of documents

In the following, we describe our approach in detail, compare it to alternative options and illustrate its embedding in a large research project.

### Process and analysis metadata: two complementary components in the specification of computational analyses

**Figure 1.**
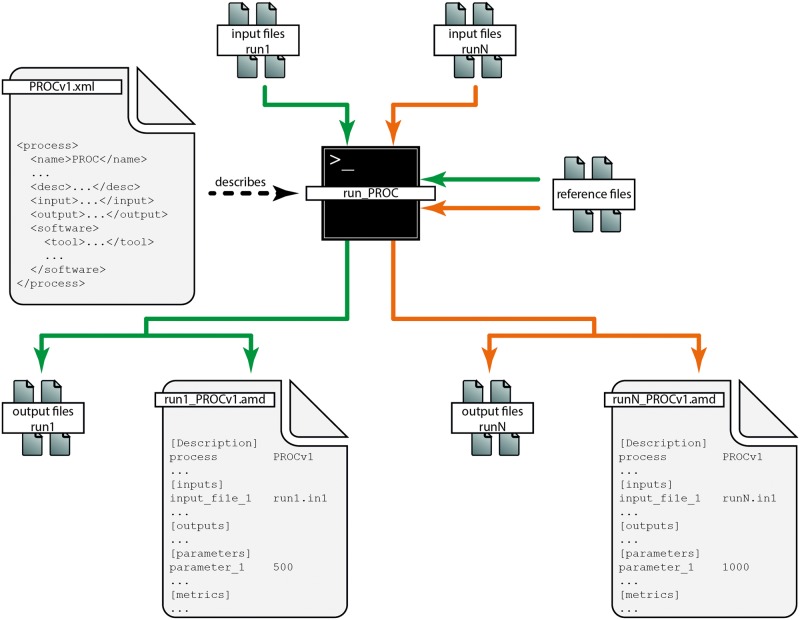
Process and analysis metadata. The basic relation between process metadata (‘PROCv1.xml’, left) and analysis metadata (‘run1_PROCv1.amd’ and ‘runN_PROCv1.amd’, bottom) is illustrated. The process describes a type of computational analysis in an abstract form, in particular specifying input (top), reference (right) and output files (bottom). The green and orange arrows represent different samples being analysed using the same process, i.e. the same sequence of software tools is applied to the input data. N analysis runs result in N distinct sets of output files (‘output files run1’ through ‘output files runN’, bottom) and N distinct AMD files containing parameters specific to the respective analysis [here exemplified with ‘parameter_1’ set to 500 (left) and 1000 (right)]. All of these N distinct AMD files link to the same process ‘PROCv1’.

Our proposal for computational metadata specification defines two complementary concepts: A ‘process’ represents the analysis steps and associated input and output files in an abstract form. An ‘analysis’ describes a realization of running such a process and is therefore unique for each execution and input (Figure 1).

#### Process metadata

**Figure 2.**
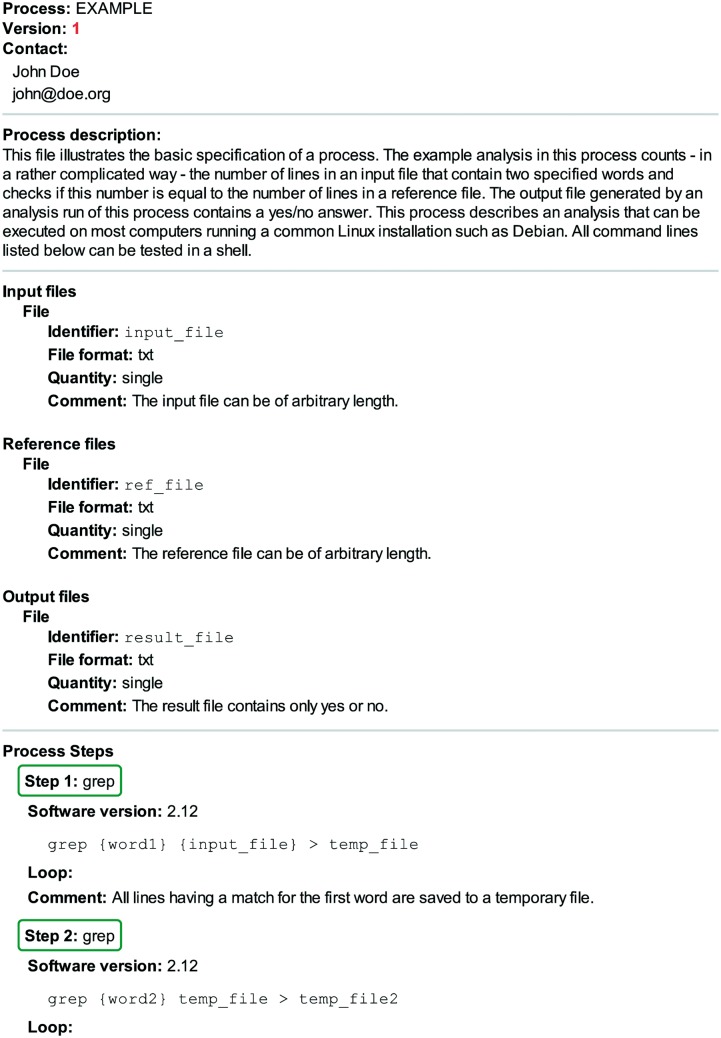
An example specification of a process. This contrived example of a minimalistic process describes a computational analysis to count lines in an input file that contain two specified words and compare the resulting number to the number of lines in a reference file (see also Supplementary File S2). This figure clearly illustrates the four main sections of a process: (i) the header containing information about the process and its author, (ii) the free-text process description, (iii) the file listings for inputs, outputs and references and (iv) the sequence of software tools (here cut for brevity, see Supplementary File S2 for the full sequence) including version information and the command line to be executed. In this example, the first command line contains the placeholders {word1} and {input_file}. Their actual values can be found in the corresponding AMD file (see Supplementary File S4).

A process (Figure 2) describes analysis steps that are applied to multiple samples or replicates of the same type within a research project. Thus a process is analogous to an experimental protocol. Typical examples in the field of (epi-)genomics include short-read alignment, ChIP-seq peak calling and RNA-seq transcript quantification. A process defines the individual steps of an analysis irrespective of the details of the sample at hand, like its species of origin, cell type or disease state. As such, a process offers an overview of how a particular file has been processed without detailing parameter settings specific to a given sample. It should provide sufficient information to enable an independent analyst (i) to grasp the overall flow of data from input to output in clearly defined steps and (ii) to execute the same analysis, with the setup of the software environment being the only major effort required. Because structure and clarity are essential in order to comply with these objectives, we decided to use XML (www.w3.org/TR/REC-xml) to specify processes. XML files are human and machine readable and can be checked for correct syntax, structure and data types against an XML schema definition (www.w3.org/TR/xmlschema-ref) file. Additionally, XML benefits from widespread software support, making it the prime choice in cases where flexible interoperability is required (10, 11).

Supplementary File S1 is an example process XML file that can be opened in a common web browser or text editor, Supplementary File S2 is the same XML with a link to a simple cascading style sheet (www.w3.org/TR/css-2010, Supplementary File S3) document, which results in improved readability when the file is opened in a web browser. The process definition comprises four sections: a header, a description, a listing of input, reference and output files and a series of analysis steps. The header section contains the process version and the contact information of the author. Incrementing the process version number implies one of the following changes to the process description: (i) the version of at least one of the software tools has been updated, (ii) the series of analysis steps has changed, i.e. tools have been removed or added, (iii) default parameters have changed for at least one of the tools or (iv) the input, output or reference file listings have changed. For instance, adding an input, reference or output file to the process should result in an increment of the version number while an updated version of reference data, such as a new genome assembly, should not, as long as the process is still executable in the same way; the information about the updated reference file would be contained in the analysis metadata (AMD) (see next section). Furthermore, improving upon or adding new comments in the process file does not require an increment in the process version.

The next section in the process XML document is a free text description of the process (see Figure 2) in which the author should outline the purpose of the process at hand. As best practice recommendation, this description should also contain details on basic assumptions or crucial computational operations. The next part of the document lists all files that are either input, reference or output files of the analysis. Here, a reference refers to a file that is used to gather additional information that are constant across many, but not necessarily all, inputs. A typical example of a reference file is the genome assembly sequence which is not specific to the analysis of a certain sample, but only changes if samples pertaining to a different genome are analyzed. We acknowledge that one might consider reference files to be just another kind of input file. However, in our experience, many scientists think distinctly about the input and the reference data in their project; a common pattern is to put input data into a different folder in the file system and not to mix it with reference data files. Each file entity has an identifier, a format, a comment field and a quantity tag. The identifier is needed for relating files to certain analysis steps. The format and comment fields entail details on the nature of a file’s content. The quantity tag can take on the values ‘single’ or ‘collection’, i.e. one or more. We give an illustrative example of the fields’ usage later in the text.

The final section sequentially lists all software tools in order of execution stating the version and the command line being executed. The command line describes the syntax for an execution in an abstract fashion, referencing file identifiers from above and containing placeholders for analysis-specific parameters in curly braces. Hence, it cannot be copied and executed as is. To make a command line executable, all placeholders have to be replaced with concrete values. These values describe an actual analysis run pertaining to a specific sample and are stored in AMD files. Furthermore, not all iterations or repetitions of certain steps, such as looping over all input files or implicit parallelization are stated explicitly but are rather indicated by a dedicated loop tag (see example below). Additionally, a comment field contains tool specific remarks such as explanations on choices for tools and parameters.

#### Analysis metadata

The metadata for a particular analysis represent an execution of a process. This implies that there is a 1:N relationship between process and AMD, where N can be large depending on the number of samples to be analyzed or due to various parameter settings that are tested for each sample (Figure 1). In contrast to the process metadata, which need to be created manually, the generation of AMD can be automated by including dedicated routines into the software that generates the analysis results. The corresponding file format should therefore be simple and well-supported by virtually all programming languages. We suggest a row oriented key-value text file, a format for which readers and writers can be implemented easily even if the programming language of one’s choice has no native support for it. Supplementary File S4 is an example AMD file that complements the corresponding process XML (Figure 2, Supplementary Files S1 and S2).

The layout of the AMD file mirrors the logical structure of the process metadata file: the first section contains general information like the process version, the date of the analysis run and the identifier of the analysis. The next section consists of three blocks naming input, reference and output files, i.e. these blocks contain the placeholders as encountered in the process metadata as keys, followed by the filenames that were part of the actual tool execution as values. Similarly, the following section contains information for the non-default parameter settings of the tools described in the process. Finally, statistics and quality metrics pertaining to the analysis run that are routinely surveyed on a per data type level are reported. One could argue that the AMD file itself is also output of the process, and as such must be included in the process XML. However, since only the joint information of process and AMD result in a complete description of an analysis run, its existence after the successful execution of the process is implicitly required.

### Example: processing and analysis of ChIP-seq data

To substantiate these generic descriptions of process and AMD files, we discuss a real world example, and elucidate their embedding in the broader context of the DEEP project as a representative of a joint research effort involving file processing and data analysis by several different institutes.

#### File listings

The ChIP-seq analysis process (CHP) we discuss here was developed in the context of the DEEP project and comprises steps for performing peak calling, for producing input-normalized signal tracks as well as generating various plots that aid in assessing data quality. It expects short-read alignment files originating from ChIP-seq experiments for multiple histone modification marks and hence supports both broad and narrow marks. Supplementary File S5 is the corresponding CHP process XML and Supplementary File S6 is its counterpart, the AMD file of an actual analysis run. Because one file per histone mark is expected, the ‘Inputs’ section of the CHP process lists standard Binary Alignment/Map (BAM) files for the histone marks as ‘collection’, i.e. one or more. Note that the input control is listed separately as ‘single’, because exactly one input control file is expected to match all histone files in an immunoprecipitation experiment. The corresponding [Inputs] block in the AMD lists the actual filenames. The BAM index files are handled in the same way, except for a comment in the CHP process stating that these files are renamed during the execution of the process to meet the naming requirements of some of the software tools used in the analysis. Based on our experience, we want to stress that such details are crucial in order to enable other researchers to run the process on their own data, avoiding a potentially frustrating trial and error phase after which the researcher might deem a process to be ‘too complicated to be used’ and instead start setting up her own, supposedly functionally identical pipeline.

The free text description of the process is the only section in the process XML that is not linked to the AMD. The ‘References’ section states that this process requires a reference genome, a file containing regions to be filtered out for certain analysis steps and a file containing genomic regions relevant for the purpose of generating quality control statistics and plots. Reference files represent static inputs for defined types of analyses. Therefore, the reference filenames listed in the AMD should always be identical when comparing compatible AMDs, i.e. AMDs for two different samples (and otherwise identical conditions) should show identical references. Analogous to the input files listed in the [Inputs] block, the [Outputs] block in the AMD lists the filename of all files that must be available after the successful analysis run. Because file naming conventions differ substantially between projects, we concede that there is probably no single best way to define an informative file naming scheme. However, based on our experience, it has proven to be useful to at least include the process identifier in every filename. This (i) links every file to a specific process and (ii) allows for shorter filenames because all necessary meta-information is stored in the corresponding, uniquely identifiable analysis and process metadata files. Specifying strict naming schemes for (output) files is particularly useful when connecting different processes as any analysis downstream of a given process in a given analysis pipeline can then in turn rely on the input files being named accordingly. Furthermore, comparing the output files generated by the analysis to the ones listed in the CHP ‘Outputs’ section allows for an easy way of checking whether or not the analysis run terminated successfully.

#### Analysis steps

The core of the CHP process details the tools and command lines for the different steps of the analysis, which in this case consists mainly of different executables of the deepTools package (12), MACS2 (13) for peak calling and two custom scripts. We examine three of the command lines in more detail to illustrate important aspects:

Step 3 (bamFingerprint tool for quality control of ChIP-seq experiments):

bamFingerprint -p {numproc} –bamfiles {GALvX_*} ––plotFile {samplesID.PROCESS.DATE.fgpplot} ––labels {labels} ––fragmentLength {all_median_fraglen} ––numberOfSamples 500 000

This command line call is an example of simple pattern matching as it is commonly used, e.g. to list filenames in a Linux shell environment. Placeholders are denoted in curly braces and it is possible to use pattern matching to reference multiple files at once. For instance, the {GALvX_*} placeholder contains the wildcard character ‘*’, indicating that this placeholder refers to all histone BAM files and the input control BAM file. There exists a variety of grammars for expressing such patterns. We deliberately advise against the use of a full-blown language for regular expressions because complicated patterns might impede human-readability of the metadata files. We suggest using simple, widely known and accepted standards as we do here. It is a valid question to ask how a user is supposed to see that this tool is executed with all BAM files at once and not with each BAM file separately, i.e. implicitly looping over all input files. In this example the loop tag is empty (see Supplementary File S5), indicating that all input files are processed at once. Another indication for this is the quantity tag of the output file with the identifier samplesID.PROCESS.DATE.fgpplot in the CHP XML, which is set to ‘single’. This implies that, irrespective of the number of inputs to the tool, there has to be exactly one file as described in the ‘Outputs’ section of the process. The {labels} and {all_median_fraglen} placeholders have no match in the process ‘Inputs’, ‘Outputs’ or ‘References’ section and thus are interpreted as a non-file parameter. Therefore, they appear in the [Parameters] block of the AMD (see Supplementary File S6). Note that the parameter ––numberOfSamples is not set dynamically, but fixed for all executions of this process to the value specified in the CHP XML.

Step 4 (computeGCBias tool to check for GC bias in the data):

computeGCBias –bamfile {GALvX_*} ––fragmentLength {*_median_fraglen} ––GCbiasFrequenciesFile {sampleID.PROCESS.DATE.gcbfreq} ––biasPlot {sampleID.PROCESS.DATE.gcbplot}

This computeGCBias call (shortened to the relevant parts) shows an example of looping. Again we find the wildcard character “*” to match all BAM files, histone as well as input control, and if one checks the process’ “Outputs” section, we see that the files identified by sampleID.PROCESS.DATE.gcbfreq and sampleID.PROCESS.DATE.gcbplot are listed with the quantity “collection”. This indicates that the number of output files changes depending on the number of input files. In contrast to the previous example, now the loop tag contains the two placeholders GALvX_histone and GALvX_input. This information is sufficient to determine which input files are part of the loop; one only has to refer to the “Outputs” section of the process to see which output file is generated for each input file. The wildcard character is also used in the placeholder {*_median_fraglen}, indicating that the AMD file must contain several entries with the suffix _median_fraglen and a prefix that is dependent on the input file, and this is indeed the case (see Supplementary File S6).

Step 5 (MACS2 tool to call histone peaks in the data):

macs2 callpeak -t {GALvX_histone} -c {GALvX_input} -f BAM –gsize {genomesize} –keep-dup all –name {*_name_prefix} –nomodel –extsize {*_median_fraglen} –qvalue 0.05 {broad}

The peak calling with MACS2 illustrates a variant of looping. As can be seen from the loop tag, the loop runs over the histone alignments, but not over the input control. This means that the input control BAM file is always the same for each execution, i.e. the input control file is kept whereas the next histone file is used. The {broad} placeholder represents a binary switch to change MACS’s behavior between broad peak and narrow peak calling. Because there is no standard way of expressing binary switches, we recommend the explicit form stating True/False in the AMD file (see Supplementary File S6, [Parameters] block). This example also illustrates a case in which the tool command line parameters do not allow to specify a full name for the output files, i.e. the parameter –name {*_name_prefix} only specifies a prefix for the output files. Hence, there is no inherent link between the command line and the output files listed in the process description. It is advisable to comment such cases in the listing for the ‘Output’ files to establish the connection. We chose to add the comment ‘Standard MACS2 output for narrow/broad marks’ for all respective output files (see Supplementary File S5).

These three examples demonstrate how to document analysis steps in terms of tool command lines containing placeholders for files or variable parameters. We intentionally refrained from developing a full-blown notational system to specify strict syntax rules for pattern matching or to distinguish between different variants of loops, as we assume that this is largely a matter of personal preference—ideally guided by common standards—or should be set in project-internal guidelines if required. Similarly, we make no assumptions how a loop is implemented, e.g. in a sequential manner or via parallelization using several CPUs or even several machines.

## Discussion

### Validation of process and analysis metadata

One substantial benefit of the XML format we chose for our metadata specification is the possibility to check the process document against an XML schema file (Supplementary File S7) in order to validate the document’s structure. Additionally, given a process and a corresponding AMD file, it is possible to automatically check for cross-references between the two files. Thus, the specification of all input, output and reference files as well as of all analysis parameters can be verified. This is a necessary precondition for turning the abstract command lines in the process XML into executable command lines. Due to the widespread support of XML and the simple data formats we propose, this automated validation procedure can be implemented in a variety of programming languages. We provide a Python 2.7/3.2 prototype implementation that is freely available (see link at the end of the text).

### Embedding and interfacing of processes

**Figure 3.**
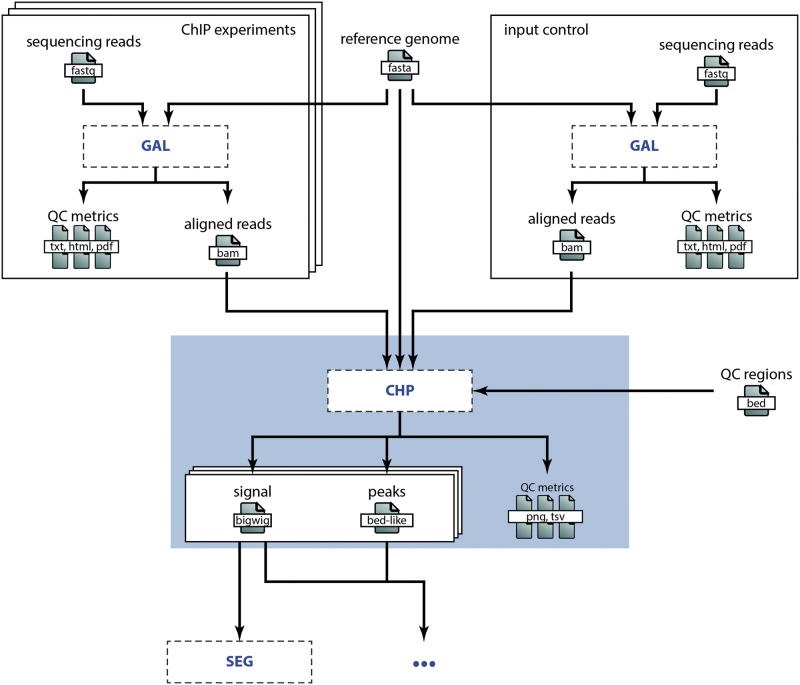
Embedding of the CHP process in a research project. This overview illustrates the embedding of the CHP process in the DEEP research project to analyze histone ChIP-seq data (omitting steps in the wet lab). The sequencing reads are aligned according to the specification in the GAL process for both the histone marks (top left) and the input control (top right). The BAM alignment files are then input to the CHP process (blue box), which is discussed in detail in the Example above. Besides these input files, the genome assembly sequence (top middle) and some annotation BED files (right) are used as reference files in the CHP process. The output files of the CHP process (signal and peaks) are then in turn input to subsequent analyses, e.g. to a chromatin state segmentation process as illustrated here.

To illustrate how different processes interface, let us consider the analysis of ChIP-seq data in a broader context: steps prior to the high-level analysis we presented in the Example involve, among others, short read sequencing, quality control and alignment. Downstream processes might encompass chromatin state segmentation [e.g. using ChromHMM (14) or Segway (15)], enrichment analyses and so on. Figure 3 shows a simplified view of the data flow for a typical ChIP-seq analysis pipeline. Because detailing all work concerning sample acquisition, preparation and sequencing is beyond the scope of this publication, we omit this ‘wet lab’ part of the data flow and start with the alignment of the raw sequencing data as specified in the GAL process (see Figure 3). The main output of this process is a set of BAM alignment files. These BAM files are then input to the CHP process, which results in the output files as described earlier. Any downstream analysis, e.g. concerning the functional characterization of the genomic regions showing histone peaks, would then be added to the chain of computational processes depicted in Figure 3. We point out that splitting the work into well-defined subtasks allows for efficient distribution of the individual steps across different research groups or institutes. Easily exchangeable and understandable annotations of computational processes are in turn pivotal to allow such a setup without a trade-off in efficiency due to communication overhead. An obvious requirement for this is an expert consensus on methods commonly used in the field. We assume that this is the case in many areas of bioinformatics research, which allows for wide applicability of our computational metadata specification.

Apart from documenting the computational analyses in a project, the generated process and AMD files can be used to build an information resource that is made public together with the actual data files. For instance, a simple website can be created, listing all files in the project and linking to the corresponding process and AMD files. Another possibility of organizing data files in relation to the process XML and the AMD is in form of entries in a database, facilitating different ways of presenting the relevant information to project partners or third parties. Because it is likely that web resources and connected databases already exist at a research institute, we refrain from making explicit suggestions about how to disseminate the computational metadata to the public.

### Related work

In contrast to existing related solutions like Galaxy (16–18) or Taverna (19), our approach does not provide immediate executability of a process (or workflow, as it is usually called in the above systems). However, this alleged shortcoming is due to the fact that the objective of our approach is a different one. Established workflow management systems commonly feature a rich graphical user interface, intuitively ‘clickable’ constructions of new workflows and easy data exchange with other instances of the same type. However, the user is required to setup the workflow inside their system, whereas our scheme is independent of operating system, internet access, preinstalled scripts or specific software packages. This is a particularly relevant concern if the user is working in an environment where they do not have full control over the software setup. Note that existing workflow management systems need to be properly installed and maintained on the production hardware (like a compute cluster) and those wrappers for all software tools typically need to be available, so that the tools can be integrated in the automated workflows. One has to keep in mind that these requirements have to be fulfilled at every location at which the processes are to be executed, which again might prove difficult if project partners rely on an already established software setup.

Our approach also affords automatic consistency checks, which is a desirable feature in large research projects to ensure consistent documentation. This is especially important as we see a shift in purpose of processes/workflows towards long-term documentation rather than allowing for immediate executability of a computational analysis. We developed our specification with the requirement of human readability, and we do not require that a particular workflow management system can now, and in five years from now, read the process XML and the AMD file (though, due to their simplicity, general machine readability can be assumed).

Another aspect concerns data management across several institutes. To the best of our knowledge, there is no software—in particular no available workflow management system—that would allow for an automated, reliable, continuous and terabyte-scale data synchronization across the heterogeneous architectures of several partner institutes. Consistent file naming is thus of high importance when data is exchanged between project partners. Our metadata specification allows for total control over filenames, which is not necessarily the case if data are put under the control of a workflow management system. A related issue is the addition or modification of reference data, which requires limited efforts in our setup, yet is something that only recently has been simplified in Galaxy (20). To summarize, in contrast to established workflow management tools, we see our approach targeted to scenarios where computational resources are distributed across independent locations, thus requiring a flexible way of defining which data is exchanged in which format, and, at the same time, can serve as full documentation of how the data was generated in the first place.

## Conclusion

We present a flexible, lightweight and modest solution to the problem of annotating computational analyses in large research projects. Our metadata specification has been developed in the context of the DEEP consortium and has proven applicable to standardizing analyses for various types of NGS assays, such as histone ChIP, DNase hypersensitivity, whole-genome bisulfite methylation and different strategies for sequencing RNA. We are confident that our work can be easily transferred to projects with other scope and needs regarding computational analyses. We think that the possibility to validate the process XML and to check it against the generated AMD file offers a new way to increase the overall quality of annotations of computational analyses. We acknowledge that certain content such as the process description cannot automatically be checked for consistency. However, this is not a problem intrinsic to our approach and, based on our experience, it can only be remedied by communication and cooperation among project partners before releasing data and annotations to the public. At the current stage of the DEEP project, many processes have been defined for default analyses and are run routinely, yet it is still to be examined to what extent integrative analyses targeting specific questions can be defined in a generalized fashion. We assume that ‘all vs. all’ or ‘disease vs. control’-types of comparative analyses or basic integrative approaches like chromatin state segmentation are feasible to describe using the current specification. Any exploratory or targeted analysis is likely to contain aspects that are in flux and related to specific biological questions and as such provides a moving target. It might therefore not be possible to specify such an analysis prior to its actual execution.

The issue of effortlessly executing a process on an independent computer is, in our opinion, not satisfactorily solvable given the current state of technology. We suppose that the concept of virtualized application containers (e.g. www.docker.com or https://coreos.com/blog/rocket) will eventually lead to out-of-the-box executable processes, freeing third-party users of the need to install and maintain a whole software environment potentially specific to a small set of problems.

We think that concisely annotated and structured computational analyses will ease reproducibility of results, offer potential to streamline complex research setups by relying on well-defined output of prior analyses and are vital to the community to understand the data published by large research consortia. Frameworks like the one described here will be crucial to achieve these goals.

## Supplementary Data

Supplementary data are available at *Database* Online.

Supplementary Data
