# Supplementary material for: A general concept for consistent documentation of computational analyses
Source: Database (Oxford). 2015 Jun 8;2015:bav050. doi: 10.1093/database/bav050 (PMC4460408; doi:10.1093/database/bav050)
Supplement: Supplementary Data [file supp_2015_bav050_index.html]

A general concept for consistent documentation of computational analyses — Supplementary Data 

# A general concept for consistent documentation of computational analyses

## Supplementary Data

files

- Supplementary Data - zip file
