# Supplementary material for: A general concept for consistent documentation of computational analyses
Source: Database (Oxford). 2015 Jun 8;2015:bav050. doi: 10.1093/database/bav050 (PMC4460408; doi:10.1093/database/bav050)
Supplement: Supplementary Data [file supp_bav050_suppl_data.zip › New Microsoft Office Word Document.docx]

**Supplementary Files**

**Supp. file 1. Process XML.** This file is an exemplary process XML file describing a contrived analysis. It is the same process as described in figure 2.

**Supp. file 2. Process XML with CSS link.** This file is identical to supp. file 1 except for a link to the CSS stylesheet (supp. file 3) and illustrates how the human readability can be improved using a basic set of layout rules as defined in the CSS.

**Supp. file 3. CSS stylesheet.** The CSS stylesheet file containing layout rules such as colors and border sizes to improve human readability of the process XML when the file is opened in a common web browser.

**Supp. file 4. Analysis metadata file.** The analysis metadata file complementing the process XML (supp. files 1 and 2) and containing actual file and parameter names for an executed analysis run.

**Supp. file 5. CHP process XML.** A real-world example of a process XML as developed in the DEEP consortium for the standardized analysis of histone ChIP-seq data.

**Supp. file 6. CHP analysis metadata file.** The analysis metadata file complementing the CHP process XML. The file contains actual file and parameters names of an executed analysis run pertaining to a mouse sample.

**Supp. file 7. XML schema file.** The XSD file defines the structure of a process XML and specifies accepted datatypes for the different fields in a process XML. It is required to validate a process XML.
